# Integrating the Human and Animal Sides of Mycoplasmas Resistance to Antimicrobials

**DOI:** 10.3390/antibiotics10101216

**Published:** 2021-10-07

**Authors:** Sabine Pereyre, Florence Tardy

**Affiliations:** 1 USC EA 3671, Mycoplasmal and Chlamydial Infections in Humans, Univ. Bordeaux, INRAE, F-33000 Bordeaux, France; 2 Bacteriology Department, National Reference Center for Bacterial Sexually Transmitted Infections, CHU Bordeaux, F-33000 Bordeaux, France; 3 UMR Mycoplasmoses Animales, Anses, VetAgro Sup, Université de Lyon, F-69007 Lyon, France

**Keywords:** mycoplasmas, antibiotic resistance, antimicrobial susceptibility testing, multidrug resistance, epidemiology of resistance

## Abstract

Mycoplasma infections are frequent in humans, as well as in a broad range of animals. However, antimicrobial treatment options are limited, partly due to the lack of a cell wall in these peculiar bacteria. Both veterinary and human medicines are facing increasing resistance prevalence for the most commonly used drugs, despite different usage practices. To date, very few reviews have integrated knowledge on resistance to antimicrobials in humans and animals, the latest dating back to 2014. To fill this gap, we examined, in parallel, antimicrobial usage, resistance mechanisms and either phenotype or genotype-based methods for antimicrobial susceptibility testing, as well as epidemiology of resistance of the most clinically relevant human and animal mycoplasma species. This review unveiled common features and differences that need to be taken into consideration in a “One Health” perspective. Lastly, two examples of critical cases of multiple drug resistance are highlighted, namely, the human *M. genitalium* and the animal *M. bovis* species, both of which can lead to the threat of untreatable infections.

## 1. Introduction

Mycoplasma is a generic term used to refer to any of the members of the class *Mollicutes*, which includes both the *Mycoplasma* and *Ureaplasma* genera [1].

The first successful cultivation of a mycoplasma was reported in 1898, unravelling the etiological agent of contagious bovine pleuropneumonia [2]. In modern human medicine, *Mycoplasma (M.) pneumoniae* was characterized as an agent of atypical pneumonia in the early 1960s and has become, by now, the most commonly studied mycoplasma species and one of the most frequent pathogens in humans, together with *M. genitalium* [3]. Mycoplasmas are isolated from a wide range of vertebrates, insects and plants, with an ever-increasing list of species [1,4]. Mycoplasmas are essentially non-zoonotic and there are only rare reports of interspecies transmissions, with a few cases of human disease due to insect-infecting *Mollicutes* of the genus *Spiroplasma*, closely related to mycoplasmas, or *Mycoplasma* spp. in immunocompromised patients [5,6,7,8], and one notable case of transmission of a caprine mycoplasma to a heavy-smoker, immunocompetent tourist visiting Cape Verde Islands [9]. Occupational infections with mycoplasmas have also been described in workers, biologists or veterinarians working with seals [10] or pigs [11]. Mycoplasmas primarily colonize and infect mucosal areas of the respiratory and urogenital tract, as well as joints, in animals and humans. Hemotrophic mycoplasmas (trivially named hemoplasmas), formerly known as *Haemobartonella* and *Eperythrozoon*, have a unique cell tropism to red blood cells [12]. Mycoplasma infection in other tissues (such as skin, central nervous system, heart, etc.) have been regularly reported. Regarding human urogenital species, extra-genital infections are usually, but not always, associated with immunodeficiency of the humoral immune response, such as a- or hypogammaglobulinemia, or immunosuppression following solid organ transplantation [13]. Brain invasion is of particular concern, as it requires a change in treatment strategy with molecules going through the blood–brain barrier [14]. *Ureaplasma* spp. have been identified as a cause of gynecologic and obstetric morbidity with associated complications in women and neonates, as well as a cause of urethritis in men. In animals, reproductive disorders associated with *Ureaplasma* spp. are infrequently reported in cattle.

The severity of clinical signs is not solely correlated with classic virulence factors, which are scarce in mycoplasmas, but also with an inappropriate host response against the infection [15], the susceptibility of the hosts being linked with abiotic factors (e.g., stress, crowding, housing conditions, climate). Mycoplasma-related infectious diseases in animals are multifactorial, often associated with other viral or bacterial infections [16].

In both humans and animals, several opportunistic or commensal mycoplasma species are frequently present in the same body niche (oropharynx, respiratory and genital tract) as recognized pathogens [4], in healthy and diseased individuals.

### 1.1. Human Mycoplasmas

Of the 18 species found in humans, only a few are clearly pathogenic and further considered relevant in this review (Table 1). *M. pneumoniae* is responsible for respiratory tract infections and some extrapulmonary complications [17]. *U. parvum*, *U. urealyticum*, (thereafter designated as *Ureaplasma* spp.), *M. hominis* and *M. genitalium* are associated with urogenital tract infections [18]. *Ureaplasma* spp. and *M. hominis* usually only colonize the urogenital tract of men and women [19] but may sometimes be responsible for infections in pregnant women and neonates or for extra-genital infections most frequently in immunocompromised patients [18,20]. On the other hand, *M. genitalium*, which is not a commensal bacterium, is responsible for sexually transmitted infections such as urethritis in men, cervicitis, pelvic inflammatory disease and adverse pregnancy outcome in women [21]. In addition, *M. penetrans* was recently sugegsted to be a new potential cause of male urethritis by analyzing the urethral microbiota of men with idiopathic nongonococcal urethritis [22], but its pathogenic role remains to be confirmed.

### 1.2. Animal Mycoplasmas

Of the 131 *Mycoplasma* species isolated from animals, a vast majority are considered as commensal (*n* = 42), opportunistic (*n* = 23), or of unclear status regarding their pathogenicity (*n* = 12); 54 are recognized as true pathogens according to the Bergey’s classification [4].

In livestock, which includes all domesticated animals raised in an agricultural setting to produce labor and commodities, such as meat, eggs, milk, fur, leather and wool (i.e., mainly cattle, small ruminants, pigs, poultry), the proportion of pathogenic species is greater (30/47) and infections are often long-lasting, resulting in significant economic losses and welfare concerns worldwide [16]. Mycoplasma infections are also rated as serious conditions in pets, while they are understudied in wildlife animals [4]. The most frequent clinical manifestations of mycoplasmosis in animals are respiratory diseases, mastitis and agalactia, arthritis, polyserositis and reproductive disorders, as well as anemia or other chronic syndromes (e.g., mild anemia, poor performance and reproductive disorders) due to the reclassified haemotrophic mycoplasmas [23].

Pathogenic species relevant for livestock are listed in Table 1. The concept of relevance takes into account the severity of the associated disease and hence its knowledge and its economic consequences, as well as the frequency of occurrence worldwide. In consequence, some haemotrophic mycoplasmas are not classified as relevant, such as *M. wenyonii* in cattle [24] and *M. ovis* in sheep and goats [25], as they are still underdiagnosed worldwide, although they certainly contribute to the general use of antimicrobials in animals.

## 2. Assumed Active Antimicrobials and Usage

### 2.1. Intrinsic Resistance

Mycoplasmas are the smallest free-living organisms characterized by a small genome, complex cultivation requirements and the absence of a cell wall [1]. The lack of a cell wall prevents them from staining by Gram stain and makes them insensitive to antibiotics targeting the cell wall, such as beta-lactams, glycopeptides and fosfomycin [26]. Mycoplasmas are also resistant to rifampicin due to an intrinsic mutation in the *rpoB* gene of RNA polymerase subunit beta, which prevents the antibiotic from binding to its target [26]. In addition, polymyxin and sulfonamides/trimethoprim are inactive due to the lack of lipopolysaccharides and the folic acid pathway in mycoplasmas, respectively [27,28]. They are also resistant to first-generation quinolones, such as nalidixic acid [29,30]. An intrinsic resistance to the group of macrolide–lincosamide–streptogramin–ketolide (MLSK) antibiotics is observed in some human and animal species. For example, among human mycoplasmas, *M. pneumoniae* and *M. genitalium* are susceptible to all MLSK antibiotics, except to lincomycin. *Ureaplasma* spp. are intrinsically resistant to clindamycin, whereas *M. hominis* is intrinsically resistant to 14- and 15-membered macrolides and to ketolides. This resistance was associated with a natural G2057A substitution (*Escherichia coli* numbering) leading to a disruption of the H bond between the 2057 and 2611 bases, resulting in an opening of the peptidyltransferase loop in the domain V of 23S rRNA [31,32]. Several animal mycoplasmas, such as *M. pulmonis*, *M. hyopneumoniae*, *M. flocculare* and *M. synoviae*, all resistant to 14-membered macrolides but susceptible to 16-membered macrolides and lincosamides, harbor the same G2057A transition [27,31].

### 2.2. Which Molecules Are Assumed to Be Active?

Antibiotics potentially active against animal and human mycoplasmas belong to MLSK, tetracyclines and fluoroquinolones. The most commonly used fluoroquinolones are not the same in human (ofloxacin, ciprofloxacin, levofloxacin and moxifloxacin) versus veterinary practice (enrofloxacin, danofloxacin and marbofloxacin) and a restricted use should be noted in animals due to the recent classification of quinolones in the list of critically important antimicrobials [33]. A few molecules active against mycoplasmas are specific to a veterinarian usage, namely pleuromutilins, such as tiamulin and valnemulin, or phenicols, such as florfenicol. Phenicols and aminoglycosides have some activity against human mycoplasmas but do not belong to the antibiotics usually used to treat human mycoplasma infections because of their toxicity and/or availability of more potent molecules. New antibiotics have been evaluated against human mycoplasmas. While pleuromutalins are often used to treat mycoplasma respiratory infections in swine and poultry, Lefamulin, a novel pleuromutilin antibiotic developed only for use in humans has recently been FDA-approved for community-acquired bacterial pneumonia with potency against macrolide-resistant *M. pneumoniae* [34]. Its activity against multi-drug resistant *M. genitalium* is also promising [35]. Eravacycline, a synthetic halogenated tetracycline derivative, also showed low minimal inhibitory concentrations (MICs) against human mycoplasmas [36]. Other antibiotics evaluated in the search for new therapeutic options against multiresistant *M. genitalium* strains include solithromycin (a fluoroketolide) [37], gepotidacin (a topoisomerase II inhibitor) [38] and zoliflodacin (a spiropyrimidinetrione) [39].

Antibiotics that result in high host-cell intracellular concentrations, such as macrolides, clindamycin and fluoroquinolones, are of particular interest, because several human mycoplasma species, such as *M. pneumoniae* or *M. genitalium*, can localize and survive within the cell [40]. Intracellular localization of animal mycoplasmas in nonphagocytic host cells has also been widely reported [41,42], suggesting the importance of using cell-penetrating antimicrobials.

### 2.3. How Are They Used?

A great majority of antimicrobial classes are used indifferently in humans and animals with a few exceptions of molecules preferentially reserved for humans (e.g., isoniazid), or limited to veterinary use due to toxicity in humans (e.g., flavophospholipols and ionophores) [43]. Some restrictions regard the patient’s age; for instance tetracyclines are contra-indicated before the age of eight in humans and fluoroquinolones are generally used after the age of 15. There is also a prioritization of use to take into account the potential rapid development of acquired resistance-associated mutations; whenever it is possible and as it is the case for standard bacteria, fluoroquinolones are preferably used as second-line treatment.

This antibiotic stewardship has been greatly encouraged lately, also in veterinary medicine, with, for instance, the enlistment of critically important antibiotics that have to be used very carefully in animals in order to limit the risk of antimicrobial resistance due to non-human use, for example colistin, fluoroquinolones or macrolides [33]. Moreover, the use of antimicrobials for growth promotion has been banned in Europe and elsewhere and phased out in some other countries, such as the United States and Canada [43]. Nonetheless, there exist main differences in chemotherapy practices between both medicines, such as individual patients’ treatments (with rare prophylactic use) in human and companion animals versus therapeutic and/or prophylactic treatment at the group level for food-producing animals (metaphylaxis) (for details see the review by McEwen et al. [43]). Group-level treatments are considered a practical and economical way to prevent spread of the infections in a herd or a lot, but they largely contribute to the antimicrobial use in livestock [44]. In animal chemotherapy, the cost of the drug is often an important criterion for choosing a treatment, ranking tetracyclines as some of the most commonly used molecules, followed by macrolides, in case of *Mycoplasma* infection [27,45].

## 3. Mechanisms of Acquired Resistance

### 3.1. Mechanisms

Target modification by chromosomal mutations is the most commonly described mechanism of resistance in mycoplasmas. Target protection by acquisition of mobile genetic elements carrying the *tet*(M) gene is limited to a few human mycoplasma species. Both mechanisms are detailed in Section 3.2.

Efflux has also been described but experimental evidence is scarce. Mycoplasma genomes are equipped with at least two classical efflux pump families, namely, the adenosine triphosphate (ATP)-binding cassette (ABC) superfamily and the multidrug and toxic compound extrusion (MATE) family [46]. However, efflux in mycoplasma has seldom been evidenced experimentally, except for fluoroquinolones in *M. hominis* [47] and *M. mycoides* subsp. *capri* [48], as well as macrolides in *M. pneumoniae* [49]. All the three studies point towards ABC-type efflux pumps, that are also able to extrude unrelated compounds, such as ethidium bromide. A recent study underlined the high inter-strain variability of efflux efficacy within *Mycoplasma* (sub)species responsible for contagious agalactia [50]. In *M. hominis*, ethidium bromide-selected strains showed a multidrug resistance (MDR) phenotype with two genes, coding for putative multidrug resistance ABC transporters, which were overexpressed [51]. Some other studies failed to demonstrate efflux [52]. In general, efflux contributes to a slight decrease of susceptibility with, for instance, only a two-fold variation of the MICs of three fluoroquinolones for *M. mycoides* subsp. *capri* [48]. This moderate impact on MICs, however, might result in selecting mutation-associated resistance, because subinhibitory concentrations of drugs are maintained within the mycoplasma cells.

Very recently and for the first time, resistance to the aminoglycosides kanamycin and neomycin by enzymatic inactivation has been described in a *M. bovirhinis* strain harboring a prophage-like region [53]. Such new findings will have to be confirmed by other groups.

Other “bypass” mechanisms leading to phenotypic resistance that have been reported in other bacterial models have been questioned in mycoplasmas, although not always formally demonstrated. For instance, persister cells organized in biofilm are known to be less susceptible to antimicrobial treatment. Biofilm-associated antibiotic resistance has been recently demonstrated in *M. genitalium* [54], *M. pneumoniae* [55] and *M*. *hyopneumoniae* [56]. As biofilm formation is being increasingly described in a number of mycoplasma species, we can further speculate that this could contribute to the development of antibiotic resistance. The intracellular location of some mycoplasmas might also limit the efficacy of non-cell penetrating antibiotics. Moreover, long term symbiosis such as the one between *Trichomonas vaginalis* and *M. hominis* might also have a slight influence on resistance [57]. Other mechanisms well-known in other bacterial models, such as small colony variants in *Staphylococcus aureus* [58], or extracellular vesicles for trapping antimicrobial or allowing spread of resistance determinants [59], remain today too speculative in mycoplasmas. Nonetheless, some unexplained increased MICs point towards other, yet unraveled, mechanisms [60,61].

### 3.2. Genetic Support

#### 3.2.1. Chromosomal Mutations

In animal and human mycoplasmas, the genetic support of resistance is mainly chromosomal mutations, which modify the antibiotic binding sites. Mycoplasmas are characterized by a high mutation frequency linked with their limited amount of genetic information dedicated to the SOS response and to DNA repair systems [62]. Notably, mycoplasmas lack the MutSLH system involved in DNA mismatch repair [1,63]. The absence of this system was associated with an increased mutation rate in other bacteria [64]. Resistance-associated mutations occur in the binding sites of the antibiotic—23S rRNA for MLSK, pleuromutilins and phenicols, DNA gyrase and topoisomerase IV genes for fluoroquinolones and 16S rRNA for tetracyclines.

##### Mutations in 23S rRNA and in Ribosomal Proteins L4 and L22

**Figure 1:**
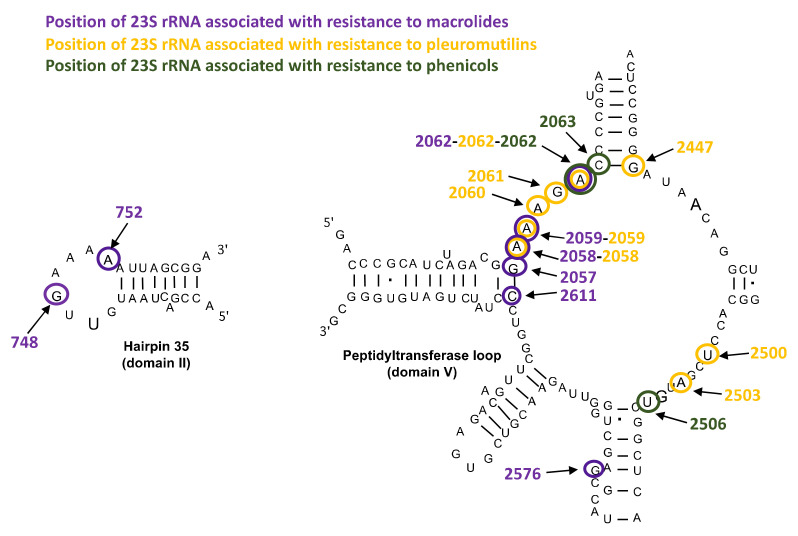
Positions of 23S rRNA mutations associated with macrolide, pleuromutilin and phenicol resistance or loss of susceptibility in human and animal mycoplasmas (*E. coli* numbering) [27,32,66,71,72].

Mutations associated with resistance to MLSK, pleuromutilins and phenicols are located in the 23S rRNA, mainly in the domain V, that includes the peptidyl transferase loop, but also in the hairpin 35 of domain II (Figure 1). The A2058G and A2059G mutations (*E. coli* numbering) are the most frequent ones and are associated with significant MICs increase in many human and animal mycoplasmas. These substitutions are responsible for cross resistance to macrolides, such as erythromycin, azithromycin, tylosin or tilmicosin, but also to lincomycin and pleuromutilins [27,65,66]. Mutations at position 2062 can be associated with resistance to macrolides, pleuromutilins and florfenicol [27,67,68]. In the domain II of 23S rRNA, the G748A substitution has been associated with tylosin and tilmicosin resistance in animal mycoplasma species only [27]. It should be noted that most *Mycoplasma* species carry only one or two rRNA operons [1]. Mycoplasmas are thus likely to develop macrolide resistance by 23S rRNA mutations because mutation of only one or two genes leads to resistance. This mechanism of macrolide resistance is unusual in bacteria harboring a higher number of ribosomal operons [69,70].

Mutations in ribosomal proteins L4 and L22 are also associated with macrolide resistance in human and animal mycoplasmas but they are only responsible for slight MIC increases and are often associated with 23S rRNA mutations [27,32,66,71].

##### Mutations in DNA Gyrase and Topoisomerase IV

Although different antibiotic molecules are used, mutations involved in fluoroquinolone resistance occur in similar, small conserved domains called quinolone resistance-determining regions (QRDRs) in both human and animal mycoplasmas. QRDRs are located in the *gyrA* and *gyrB* genes, encoding the subunits A and B of the DNA gyrase and in *parC* and *parE* genes encoding the subunit C and E of the topoisomerase IV, respectively [26]. However, as for classical bacteria, the primary mutation target (either DNA gyrase or topoisomerase IV) varies according to the mycoplasma species and to the fluoroquinolone drug. In the human species *M. genitalium*, *Ureaplasma* spp. and *M. hominis*, the *parC* gene is the most frequently altered in resistant clinical isolates (mainly amino acid positions 80 and 84, *E. coli* numbering) [65,73]. Mutations in *gyrA*, *gyrB* and *parE* genes were also reported in clinical isolates, but were usually either associated with a *parC* mutation or associated with lower MIC increase. In animal mycoplasmas, the most frequent point mutations leading to significant increase in MICs were observed in *gyrA* (amino acid positions, in *E-coli* numbering, 81 and 83, as well as 84 and 87, albeit less frequently) and *par*C (positions 80, 81 and 84). Mutations in *gyrB* and *parE* were observed only in in vitro selected mutants of *M. agalactiae* and *M. gallisepticum* (for a review see [27]). The combination of mutations within DNA gyrase and toposiomerase IV leads to highly resistant isolates in both animal and human mycoplasma species [27,65,73].

##### Mutations in 16S rRNA

**Figure 2:**
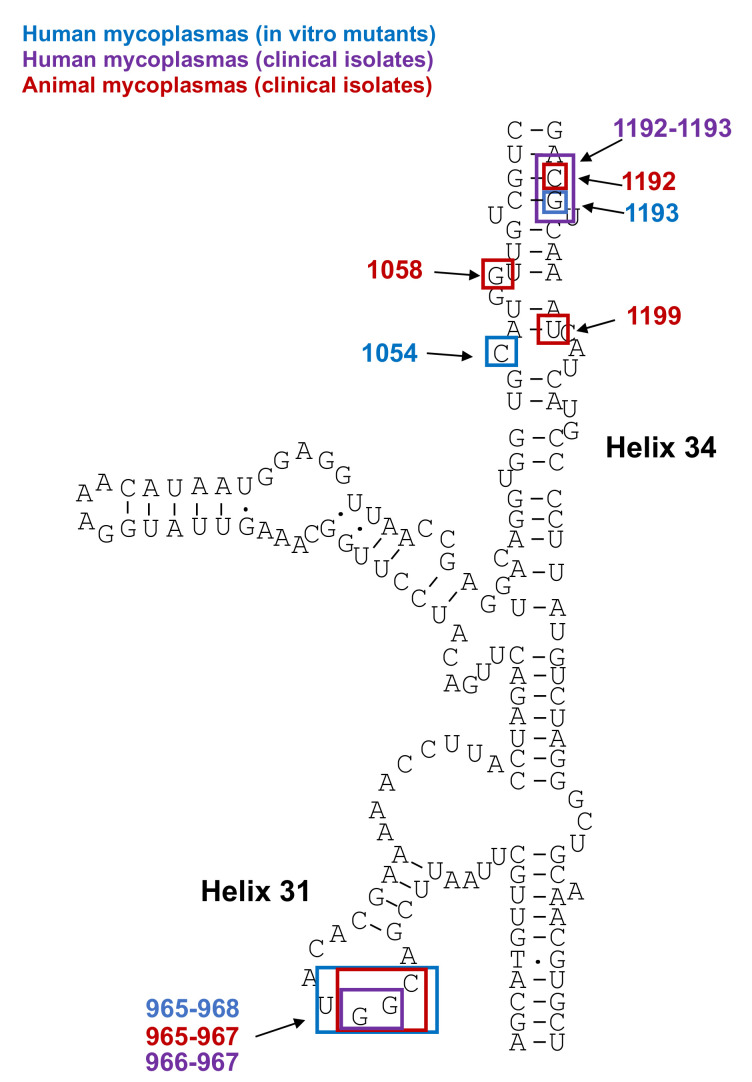
Positions of 16S rRNA mutations associated with tetracycline resistance or loss of susceptibility in mycoplasmas (*Escherichia coli* numbering) [27,74,75,76].

The tetracycline binding pocket is composed of helixes 31 and 34 of the 16S rRNA genes and mutations within these helices can result in increased tetracycline MICs in mycoplasmas [26]. The main mutation site is located in helix 31 at positions 965, 966, 967 and 968 (*E. coli* numbering), but positions 1054, 1058, 1192, 1193 and 1199 were also reported [74,75,76] (Figure 2). In human mycoplasmas, these mutations were mainly selected in vitro and were only associated with a slightly reduced susceptibility [74]. Indeed, no tetracycline-resistant isolates have been reported through mutations in human mycoplasma species to date. Mutations in the 16S rRNA gene were recently reported in clinical isolates of *M. genitalium*, but their involvement in tetracycline resistance remains to be demonstrated [75,76]. In contrast, in animal mycoplasmas, field isolates highly resistant to tetracycline were reported in *M. bovis*, with both ribosomal operons being mutated at two or three positions [77,78]. Some isolates with increased MICs were also described for *M. agalactiae* [61].

#### 3.2.2. Acquisition of Mobile Genetic Elements

In the urogenital human mycoplasmas *Ureaplasma* spp. and *M. hominis*, but not in other human or animal mycoplasma species, the *tet*(M) gene is responsible for high-level resistance to tetracyclines [72]. The Tet(M) protein shows homology with the elongation factors EF-Tu and EF-G. It binds to ribosomes conjugated with tetracycline and induces permanent conformational changes of the ribosome that ejects the tetracycline molecule from the ribosomal complex and prevents re-binding of the antibiotic without altering protein synthesis [79,80]. The Tet(M) protein confers cross-resistance to all tetracyclines with MICs over 8 µg/mL. Glycylcyclines, such as tigecycline, retain activity against *M. hominis* carrying the *tet*(M) gene [81]. *Ureaplasma* spp. are intrinsically less susceptible to tigecycline than to tetracycline and minocycline, but, in the presence of the *tet*(M) gene, the increase of tigecycline MIC is less pronounced than that of tetracycline and minocycline [81]. The *tet*(M) gene is inherited by horizontal gene transfer via transposons, with transposon Tn*916* associated with its dissemination in mycoplasmas. In the *M. hominis* Sprott isolate, it was shown that a truncated Tn*916* resides within an uncharacterized transposon, closely related to transposons from streptococci [82]. The absence of Tn*916* conjugation genes precludes mobility, but the larger mosaic element retains competency for excision and circularization. It was subsequently shown that, in several *M. hominis* strains, this *tet*(M)-harboring transposon of variable length comprises a 13 kb region homologous to Tn*916* and is consistently integrated between the somatic *rumA* gene and an hypothetical protein [83,84].

The *tet*(M) carrying Tn916 transposon is a notable exception, as there have been a limited number of mobile genetic elements in mycoplasmas described so far. Whereas no plasmids have been detected to date in human mycoplasmas, only a few small and cryptic plasmids have been reported in ruminant pathogens of the *M. mycoides* cluster, in the plant pathogen *Spiroplasma citri* and in several phytoplasmas [85]. Mycoplasma integrative and conjugative elements (MICE) have also been reported in 14 different human and animal species that belong to two phylogenetic groups, namely, Hominis and Spiroplasma [86]. MICEs are large modular chromosomal regions of 22–37 kb that encode for about 20 structural genes flanked by two inverted repeats, with a structural gene at their 3′ end that encodes a DDE recombinase. Entire functional MICEs randomly inserted in the chromosome often occur in multiple copies in a single mycoplasma genome, along with MICE vestiges, raising the question of the cost of these large elements on the fitness of small genome mycoplasmas. To date, MICEs have not been shown to harbor any cargo genes associated with antibiotic resistance. Although MICEs have also been reported in about 45% of *M. hominis* clinical isolates, they are not likely to be associated with tetracycline resistance [87].

Contrary to early dogmas, gene losses are not the only scenario driving mycoplasma evolution and horizontal gene transfers (HGT) have been shown to contribute to shaping current mycoplasmas genomes, enriching them in genomic islands, such as phages and MICE [86]. However, except for *tet*(M) in *M. hominis* and *Ureaplasma* spp. and the recently reported antibiotic inactivating enzyme genes in *M. bovirhinis* [53], no other mobile genetic elements have been identified as a carrier for antimicrobial resistance. Hence, the contribution of HGT to antimicrobial resistance was first thought to be minor, in mycoplasmas. However, the recent demonstration of an ICE-dependent, unconventional conjugative mechanism providing susceptible mycoplasma cells with the ability to rapidly acquire, from pre-existing resistant populations, multiple chromosomal loci carrying mutations responsible for antimicrobial resistance might make a difference [88]. This HGT mechanism, known as mycoplasma chromosomal transfer (MCT), uses the MICE-dependent conjugation machinery between two cells to generate fluxes of large portions of the chromosome, from the MICE-negative to the MICE-carrying strain. Subsequent homologous recombination events result in generating chimeric genomes, the transfer of nearly every position of the mycoplasma chromosome being possible. Through opening the possibility of a one-step transfer of several point mutations associated with resistance, MCT could act as accelerator for antimicrobial resistance dissemination. However, whether this could play a role in vivo in the emergence of antibiotic resistance in pathogenic mycoplasmas has yet to be demonstrated. Transconjugants were obtained in vitro only through using antibiotics as selective pressure, but there is, so far, no further demonstration of the viability, fitness of the resulting mosaic genomes, or their adaptability in natural environment [88].

The recent possibility of transmission of resistance genes through extracellular vesicles [89] requires further experimental evidence in mycoplasmas and hypothesis regarding the integration of the modified gene. This has been demonstrated in other bacterial genera but might be limited to antimicrobials acting on membranes [59,90,91].

## 4. Susceptibility Testing and Epidemiology of Resistance

### 4.1. Methods for Determination of Antimicrobial Susceptibility

#### 4.1.1. Phenotypic Methods

Agar disk dilution should never be used for antimicrobial susceptibility testing (AST) of mycoplasmas, because there are no data to correlate the inhibition- zone diameters with MICs and also because of the complexity of the medium, resulting in a non-linear diffusion of the antimicrobial from the disk. In 2011, the Clinical and Laboratory Standards Institute (CLSI) subcommittee on antimicrobial susceptibility testing of human mycoplasmas published media and consensus methods for implementation and quality control of antimicrobial testing of *M. pneumoniae*, *M. hominis* and *Ureaplasma* spp. using the broth microdilution and agar dilution techniques, along with the corresponding clinical breakpoints (CBPs) to determine susceptibility or resistance [92,93]. Several commercial kits using the broth dilution technique are available in Europe for susceptibility testing of *M. hominis* and *Ureaplasma* spp. but not of *M. pneumoniae* [94]. These kits consist of microwells containing dried antimicrobials, in one or two concentrations, corresponding to the CLSI clinical breakpoints for the most recent ones. Performance of some of them have been evaluated in comparison to the reference CLSI methods [73,95]. It should be noted that, for *M. genitalium*, such phenotypic susceptibility testing methods are not practicable because its growth is too fastidious.

Phenotypic methods for animal mycoplasmas are largely lagging behind that of human ones. Despite a first robust AST methodology based on MIC determination proposed in 2000 by Hannan [96], there is still a need to better standardize veterinary mycoplasma AST by developing harmonized methodologies (quality controls, reference strains, etc.) and interpretative criteria, i.e., CBPs. Updated recommendations for AST are provided in the recent review by Bouchardon et al. [27]. CBPs are hard to establish in veterinary medicine, as they need to be species-specific, substance-specific and disease-specific [97]. Because of these multiple combinations of animal/bacteria species and clinical conditions, as well as variations between the nutritional requirements and fitness between species, human CBPs cannot be used for animal mycoplasmas. Initiatives such as the ENOVAT cost program are expected to define CBPs for several bacteria of veterinary importance (https://enovat.eu, accessed on 1 September 2021). In the meanwhile, epidemiological cut-off values (ECOFFs), i.e., the highest MIC that defines the upper end of the wild-type (WT) MIC distribution, could be used as surrogates, i.e., thresholds for early warning of acquired phenotypic resistance, but not for guiding therapy, as non-WT isolates are not always clinically resistant. Furthermore, the process of setting ECOFFs is also challenging, as, according to the European Committee on Antimicrobial Susceptibility Testing (EUCAST, https://www.eucast.org, accessed on 1 September 2021) standard operating procedures, it requires aggregation of MIC data obtained in different laboratories using standardized AST methods [98]. Today, if more and more results are regularly published, they are hardly comparable, even when comparing MIC data, as there might be an influence of the method [27]. The absence of standardized methods suitable for all animal species also prevents the development of commercial kits. For instance, 96-well Sensititre plates need to be customized to contain the right antimicrobials at the right concentrations [99]. Some studies also suggested the use of an antibiotic gradient strip for fast-growing mycoplasma species, either from humans (*M. hominis*) or animals (*M. bovis* and *M. agalactiae*), but those have never been standardized [100,101,102].

#### 4.1.2. Genotypic Methods

Due to the rise in macrolide resistance in two human fastidious species, *M. pneumoniae* and *M. genitalium*, nucleic acid amplification tests have been developed for the detection of mutations associated with macrolide resistance. Several in house real-time PCR with different technologies, such as TaqMan assay, melting-curve analysis or pyrosequencing, were first developed for both species [66,103,104,105]. The advantages of these methods are that they can be used directly on the primary clinical specimens and avoid the need for fastidious and time-consuming growth of the bacteria.

During the last five years, several commercial kits have been launched in Europe and have shown good sensitivity and specificity for the simultaneous detection of *M. genitalium* and from four to six macrolide resistance-associated mutations [106,107,108]. These commercial developments were pushed forward by a growing demand from clinicians in the absence of any culture possibility together with an increasing resistance prevalence. In contrast, fluoroquinolone resistance-associated mutations in *M. genitalium* are still mainly searched by amplification and sequencing of the target genes. A few attempts to develop commercial kits that directly detect several mutations in the *parC* gene were published [109,110], but the correlation between certain single nucleotide polymorphisms (SNP) and MICs was not established, jeopardized by the fastidious growth of the bacteria. Additionally, some reported SNPs seem not to be associated with treatment failure [111,112].

SNP genotyping using Melt Analysis of Mismatch Amplification Mutation Assays (Melt-MAMA) has also been proposed for detection of resistance-associated mutations in animal mycoplasmas, but it is usually based on in-house tests, that have never been validated on a large scale [113,114]. For *M. bovis,* a good correlation between melting-profiles and MICs has been noted for several antimicrobials [113], but not for *M. synoviae*, due to the existence of numerous non-hotspot mutations in the target genes [114]. The method is not applicable on all the antimicrobials and is highly dependent on the PCR machine used. In addition, some initiatives for other PCR methods (Taqman SNP real-time PCR assay) have been published but never adopted by the diagnosis community [115].

### 4.2. Epidemiology of Resistance

#### 4.2.1. Human Mycoplasmas

##### Prevalence of Resistance in *M. pneumoniae*

To date, no tetracycline or fluoroquinolone resistance has been reported in clinical isolates of *M. pneumoniae*. Prior to the 2000s, very few *M. pneumoniae* clinical isolates were resistant to macrolides. In Japan, a constant increase in macrolide resistance rates was then reported until 2011, reaching 30% in 2006, 60% in 2009 and around 90% in 2010–2011, with regional rate differences [66]. A decrease in macrolide-resistant strains down to 11% was recently reported in this country [116]. This change was associated with a decrease in macrolide consumption and possibly with a shift in the prevalent genotype of *M. pneumoniae*, from the macrolide-resistant adhesin P1 type 1 before 2011 to the adhesin P1 type 2 harboring no macrolide resistance-associated mutations [117]. In China, prevalence of macrolide resistance reached 100% [118], linked to the extensive macrolide use in this country. In contrast, in North America, Europe and Australia, macrolide resistance rates remained lower, usually not exceeding 12% [66,119,120,121]. Because of contra-indication of tetracyclines and fluoroquinolones in young children, *M. pneumoniae* antibiotic treatment may be hampered in countries with high macrolide resistance rates. If available, tosufloxacin, a fluoroquinolone approved for children in Japan, represents an alternative treatment [122].

##### Prevalence of Resistance in *Ureaplasma* spp. and *M. hominis*

In contrast to *M. pneumoniae*, tetracycline and fluoroquinolone resistance is commonly reported in *Ureaplasma* spp. and *M. hominis*, whereas macrolide resistance is very rare, limited to a few case reports. In France, prevalence of fluoroquinolone resistance was low in *Ureaplasma* spp. and *M. hominis*, reported at 1.2% and 2.7% for levofloxacin and 0.1% and 1.6% for moxifloxacin, respectively, between 2010 and 2015 [73]. Considering only studies which used the reliable reference microdilution broth assay from CLSI [93], the rates of levofloxacin-resistant *Ureaplasma* spp. were 0% between 2007 and 2013 in England and Wales [123], 0.54% between 2017 and 2018 in Wales [124], 1.4% between 2001 and 2006 [125] then 6% between 2015 and 2016 in the USA [126], but 57% between 2007 and 2013 in Japan [127], and 47.5% in 2017–2018 [128] and 84.3% in 2018 in China [129]. Regarding tetracycline resistance, no clinical resistance through the 16S rRNA mutation has been reported in human species and all resistant isolates harbored the *tet*(M) gene. A recent meta-analysis found a midrange resistance rate for *Ureaplasma* spp. and *M. hominis* to tetracycline of 43.3% and 50%, respectively, but a high level of heterogeneity was observed among studies [130], with potential overestimation of resistance due to the use of commercial kits [73,95]. Considering only studies which used the reference microdilution broth assay, the rate of tetracycline resistance in *Ureaplasma* spp. and *M. hominis* was 7.5% and 14.8% between 2010 and 2015 in France and 0.5% and 2% in 2017–2018 in Wales [124], respectively. In reports that only studied *Ureaplasma* spp. susceptibility, resistance to tetracycline was 2.3% between 2007 and 2013 in England and Wales [123], 1.4% between 2001 and 2006 in the USA [125] and 19.7% in 2017–2018 in China [128].

##### Prevalence of Resistance in *M. genitalium*

See Section 5.1 below.

#### 4.2.2. Animal Mycoplasmas

According to Sweeney et al. [131], the term ‘resistance’ should be reserved for “situations that have clinical implications for a patient”, meaning that the clinical outcome of treatment should be taken into consideration. Hence, it is dependent on the definition of species-specific CBPs. As a consequence, in the absence of CBPs, the definition of a resistant population is compromised in animal mycoplasmas. Three strategies are most often used to address this issue. Those are (1) using ECOFFs as surrogates to CBPs, when a coherent WT population is defined; (2) using CBPs from another bacteria colonizing the same ecological niche in the same animal (for instance the CBPs of respiratory *Pasteurellaceae* used for *M. bovis*, as they share a common tissue tropism in the bovine host); (3) using non-species-specific (mostly human) interpretive criteria. None is ideal as none takes into account the potential impact of interspecies pharmacokinetics on clinical outcome.

It was suggested that the term ‘non-susceptibility’ should be used instead of resistance, including more largely resistant, intermediate or non-susceptible populations [132]. In any case, the methods and interpretive criteria used should be explicit in any reports of future surveillance in the veterinary field. Some data, obtained with different methodologies, are sufficiently coherent to achieve consensus but most often the geographical overall picture of “resistance” prevalence is partial.

In ruminants, mycoplasmas are mostly susceptible, with a few high-MIC isolates, except for *M. bovis*, that could be considered as multiresistant worldwide, according to the definition of Magiorakos [27,98,132,133]. The multiresistance pattern of *M. bovis* is detailed in Section 5.2. As for the etiological agent of contagious bovine pleuropneumonia, an Office International des Epizooties-listed disease, recent data are scarce. Indeed, only a few isolates of *M. mycoides* subsp. *mycoides* have been tested for their antimicrobial profile. They were mostly susceptible with some emerging resistance detected in the early 2000s [134]. Although antimicrobial treatment is officially discouraged, as it may alleviate the clinical signs while not preventing the spread of infection and favoring the creation of chronic carriers, it remains a current practice in Africa [135].

In poultry, some species, such as *M. meleagridis* and *M. iowae*, are understudied and no general trends about their susceptibility pattern can be proposed. *M. gallisepticum* has remained mainly susceptible to most molecules, namely, tiamulin, florfenicol, tetracyclines, macrolides and fluoroquinolones, although some high prevalence (50–70%) of resistant strains to the latter two antibiotic families have been described in Israel [136,137]. *M. synoviae* is, similarly, mostly susceptible to tiamulin and tetracycline, but several increased MICs of fluoroquinolones and macrolides have been reported in different countries (for a recent review, see [27]).

In swine, most antimicrobial families are very active against *M. hyopneumoniae*, especially pleuromutilins [27]. Some resistant isolates have been described for fluoroquinolones and MLSK [27].

## 5. Critical Cases of Multiple Drug Resistance: Examples of Two Mycoplasma “Super” Bugs, *M. bovis* and *M. genitalium*

### 5.1. M. genitalium

*Mycoplasma genitalium* is an emerging genital mycoplasma responsible for sexually transmitted infections, discovered in 1980 [138]. *M. genitalium* is a cause of nongonococcal urethritis [139] in men, cervicitis and pelvic inflammatory disease in women [140] and may be associated with preterm birth and spontaneous abortion, but more studies are needed to confirm its role [140]. It is noteworthy that more than 50% of infected patients, men and women, remain asymptomatic [21,141].

*M. genitalium* prevalence ranges between 1 and 2% in the general population in countries with higher levels of development [142,143,144,145] and 3.9% in countries with lower levels [146], but can reach up to 30% in high sexual-risk population [21,147,148].

The American Center for Disease Control and Prevention mentioned *M. genitalium* in the watch list of antibiotic-resistant bacteria, based on the level of concern to human health, in its 2019 antibiotic resistance threat report (http://www.cdc.gov/drugresistance/Biggest-Threats.html, accessed on 1 September 2021). Treatment options are limited, with a noticeable poor efficacy of tetracyclines, as doxycycline can only eradicate *M. genitalium* from 30% of infected patients [21], despite a relative potency in vitro. The macrolide azithromycin is the first-line treatment recommended by the European guideline on *M. genitalium* infections, with an extended course of 5 days (500 mg on day one, then 250 mg on days 2–5) [21]. High-level resistance to macrolides is associated with point mutations at positions 2058, 2059 and, more rarely, 2062 in region V of the 23S rRNA gene [68,72]. In France, there was no macrolide-resistant *M. genitalium* strains before 2006 [149]. Between 2006 and 2014, macrolide resistance was quite stable, ranging between 10% and 17% [149,150], but is now higher than 35% [151]. The same trend has been observed all over the world. A recent meta-analysis reported a summary prevalence of macrolide resistance-associated mutations of 35.5%, with a prevalence that increased from 10.0% before 2010 to 51.4% in 2016–2017 [65]. However, significant difference of prevalence was observed according to the gender and the sexual behavior of patients, with the highest prevalence reported among men who have sex with men [151,152,153,154]. High prevalence of macrolide resistance in *M. genitalium* was shown to be correlated with macrolide consumption in 18 countries [155]. Finally, because of the high macrolide resistance in *M. genitalium* worldwide, it is now recommended that all *M. genitalium*-positive tests be followed with an assay detecting macrolide resistance-associated mutations [21,156], to enable rapid choice of an effective first-line antibiotic therapy.

Moxifloxacin and sitafloxacin are the only fluoroquinolones active against *M. genitalium,* as other fluoroquinolones have too-high MICs [18]. Moxifloxacin is the second-line treatment recommended in most countries in case of uncomplicated *M. genitalium* infection known or suspected to be macrolide-resistant, or as a first-line treatment in case of complicated infection, such as pelvic inflammatory disease [21,156] (http://www.sti.guidelines.org.au/sexually-transmissible-infections/mycoplasma-genitalium, accessed on 1 September 2021). Resistance to moxifloxacin is associated with mutations in the QRDR region of the topoisomerase IV gene *parC*, which primarily affects amino acid positions Ser83 and Asp87 (*M. genitalium* numbering, corresponding to positions 80 and 84 in *E. coli*). Although several mutations have been reported in the *parC* gene, only a few have been confirmed to be associated with increased MICs and/or moxifloxacin treatment failure, namely, Ser83Ileu, Ser83Arg, Asp87Asn, Asp87Tyr and Gly81Cys [157,158,159,160]. Mutations in the DNA gyrase *gyrA* gene, such as Met95Ileu and Asp99Asn (*M. genitalium* numbering), have also been reported but are generally associated with a ParC mutation. Treatments were more likely to fail if concurrent ParC and GyrA mutations were present, suggesting an additive effect [157]. In a meta-analysis, the overall prevalence of fluoroquinolone resistance-associated mutations was 7.7% and did not change significantly over time [65].

In several studies, a large distribution of *M. genitalium* genotypes was found among macrolide- and fluoroquinolone-resistant isolates, supporting the hypothesis of a multiclonal spread of resistance in this species, rather than the spread of a single or of a few resistant clones. This multiclonal spread is likely associated with the consequences of antibiotic selection pressure on diverse independent *M. genitalium* strains [161,162,163].

Dual macrolide and fluoroquinolone resistance was reported in 2.8% *M. genitalium*-positive samples [65], with prevalence up to 30.8% in Japan [164] and 87.7% in men with symptomatic urethritis in China between 2011 and 2015 [165]. Such dual resistance is mainly due to successive treatment failure of macrolides then fluoroquinolones. After azithromycin and moxifloxacin failure, few therapeutic options remain available. According to the definition of extensively drug-resistant (XDR) bacteria proposed by a group of international experts in 2012, such *M. genitalium* isolates could be categorized as XDR because they remain susceptible to only one or two antimicrobial categories [132]. A doxycycline third-line treatment can be tried, but only 30% of the patients are cured [21]. Pristinamycin, a streptogramin combination, can also be used in countries where it is available, but high doses must be used, exposing patients to side effects [21]. To improve the efficacy of antimicrobial treatment, a resistance-guided sequential therapy was recommended in Australia and the UK [156] (http://www.sti.guidelines.org.au/sexually-transmissible-infections/mycoplasma-genitalium, accessed on 1 September 2021). This sequential therapy relies on the initial use of doxycycline for 7 days, immediately followed by administration of azithromycin or moxifloxacin, depending on the macrolide susceptibility of the *M. genitalium* strain. The bacterial load was shown to be decreased after the first week of doxycycline administration [166,167], improving the efficacy of the second antimicrobial. In addition, early data on combination therapy with doxycycline and sitafloxacin, or with doxycycline and pristinamycin, also showed promising results [168,169]. Other therapeutic options include minocycline, which cured 71% of *M. genitalium* infection cases in a recent report [169] and spectinomycin, in countries where it remains available, which showed clinical success after several antibiotic failures [170].

Overall, only a few molecules are potent against *M. genitalium* and, because of the decline in efficacy of available antibiotics, the threat of untreatable *M. genitalium* infections is now getting closer.

### 5.2. M. bovis

The first report of *M. bovis* infection dated back to 1961 from a mastitis case in the USA [171]. Almost 60 years later, *M. bovis* has now a largely described global distribution with a recent introduction in countries such as Finland and New Zealand, which had been, so far, free of this species [172]. It has been associated with different clinical manifestations, such as pneumonia (as one of the etiological agents of the bovine respiratory disease, BRD), mastitis, arthritis, otitis media and other less frequent disorders, such as keratoconjunctivitis, meningitis, cardiac diseases, or genital disorders [173]. It is considered to be one of the major emerging pathogens of cattle in industrialized countries threatening livestock production [174] and accounting for significant economic and production losses in the beef and dairy industries [173]. The epidemiological picture of *M. bovis* infection is not homogeneous worldwide due to differences in breeding density and practices.

In the absence of universally available vaccines [175], sanitary control measures and chemotherapy are the only methods to control *M. bovis* infections. Treatment practices also differ greatly between the different countries. For instance, in North America, drugs such as tulathromycin are used not only for therapy but also for metaphylactic injections on calves’ arrival at the feedlot [176]. Overall, the most often used antimicrobials against *M. bovis* worldwide are tetracyclines (oxytetracycline), macrolides (gamithromycin, spiramycin, tilmicosin, tulathromycin and tylosin) and fluoroquinolones (danofloxacin, enrofloxacin and marbofloxacin). In Europe, amphenicols (florfenicol) are also licensed [177]. Macrolides, known to concentrate in the lungs and to penetrate into the host cells, are a good option, notably for respiratory infections.

Since 2000, reports indicating that *M. bovis* has developed multiple antimicrobial resistance over time have started to accumulate. This multiresistance pattern is supported by both phenotypic data (high MICs; for a recent review, see [27,178]) and increased evidence of resistance-associated mutations [60,77,78,179,180,181], consistently with reports of treatment failure. However, depending on the studies, the proportion of *M. bovis* isolates with decreased susceptibility is highly variable, resulting in a wide range of MICs in reviews. This might reflect the year of sampling, the country of origin with its own regulatory practice for use and feedlot management practices, the type of livestock production system, the infection localization, etc. However, the susceptibility patterns might also differ, owing to the methodology used to measure MICs, which shows an unfortunate bias due to the absence of harmonization. For instance, in a recent study using Sensititre plates, MICs of florfenicol and oxytetracycline for recent Canadian isolates were lower than those reported in 2010–2012 French isolates, whether MICs for macrolides (except tilmicosin) were higher [176]. Whether this is due to methodology bias, or to true difference in the susceptibility of isolates, is yet to be resolved. Only differences observed within a single study can be interpreted. For instance, tulathromycin frequently used to treat respiratory infection and with no marketing authorization for mastitis has higher MICs for respiratory isolates versus mastitis ones [182] and reciprocally for spectinomycin; lung isolates have lower MICs than mastitis isolates [183]. Since 2010, the MycoPath project, a pan-European program dedicated to the collection and monitoring of antimicrobial susceptibility of veterinary mycoplasmas from diseased food-producing animals not recently exposed to antimicrobials, was set up. This project is coordinated by the Executive Animal Health Study Center (CEESA) in Brussels, whose members are mainly international pharmaceutical companies. Isolates collected all over Europe are centralized into one central lab that carries out MIC testing, reducing the risk of inter-laboratory test variations. Because of sufficiently large test populations, MycoPath results are expressed using MIC_50_ and MIC_90_ values, which represent the MIC values at which ≥50% and ≥90% of the isolates are inhibited, respectively. The latest report [177], showed high homogeneous MIC_90_ values for *M. bovis* with minor variations between countries, with global MIC_90_ values of 16 mg/L for spiramycin; >64 mg/L for tylosin, gamithromycin, tilmicosin and tulathromycin; 8 mg/L for florfenicol; 32 mg/L for oxytetracycline. For fluoroquinolones, the MIC_90_ were more variable between countries with higher values in Italy and Spain, where this drug family has been more widely used in the past [177]. Within the CoVetLab consortium (https://www.covetlab.org/c5, accessed on 1 September 2021), we conducted a monocentric study with isolates from Nordic European countries that reached the same conclusion, that is, recent isolates shared the common feature of lowered susceptibility to major antimicrobial families, such as macrolides, tetracyclines and phenicols, but a preserved susceptibility to fluoroquinolones and aminoglycosides [172].

These high-MIC data, even in the absence of CBPs, point towards a multi resistance pattern. In France, the substantial increases in macrolide MICs (tylosin, tilmicosin, tulathromycin, tetracyclines, etc.) of isolates collected within a 30-year interval [27] was attributed to the spread of a single clone from the year 2000 onwards, showing a homogeneous genotype of resistance [78,184]. Spread of a homogenous clone in Denmark which has subsequently emerged in Sweden and Finland has also been recently suggested [172]. In other countries, the situation can be different and an increase in resistance is not always associated with a loss of genetic heterogeneity [185].

It should be noted that the capacity of in vitro susceptibility testing to predict clinical efficacy of drugs has been largely controversial for *M. bovis*, due to the limitations we have already mentioned for animal mycoplasmas, i.e., no harmonized methodology for AST and no pharmacokinetics (PK)/pharmacodynamics (PD) data to interpret MICs. It was shown, in an industry-sponsored study, that tulathromycin was efficacious in treating calves infected with a strain of *M. bovis* that had MICs of >64 mL/mL for tulathromycin [186]. Whether this could be due to the anti-inflammatory properties of macrolides by suppressing the “cytokine storm” has yet to be demonstrated [187].

## 6. Conclusions

Mycoplasmas are not an exception to the general trends of increasing antimicrobial resistance worldwide. However, the phenomenon is highly variable in terms of MIC values attained, but also in terms of prevalence, depending on the species. The contribution of mycoplasmas to the global resistance-associated gene flux between different bacteria genera is poor, as the genomic support of resistance is essentially chromosomal point mutations, except for the *tet*(M) gene in *M. hominis* and *Ureaplasma* spp. in humans. Nonetheless, in a “One Health” perspective, resistance in mycoplasmas has to be taken into consideration, as it impacts the use of antimicrobials. In certain animal and human mycoplasma species, the identification of new drugs is needed, but many will not be available for some time. In the meanwhile, new treatment modalities, such as combination therapies, sequential therapies or resistance-guided therapies could be assessed, as we have already started to struggle with some multi-drug resistant *M. genitalium* strains.

This review clearly emphasized several gaps within human and/or veterinary medicines regarding the current knowledge on antimicrobial resistance in mycoplasmas:

- A lack of harmonized methodologies for AST of animal mycoplasmas, as well as the absence of clinical breakpoints, preventing data interpretation.
- The unavailability of European clinical breakpoints for human mycoplasmas that might be more adapted to European practices
- A lack of antibioresistance data on some specific mycoplasmas of cats, dogs and horses, as well as on non-cultivable haemotrophic mycoplasmas.

Last but not least, data about the fitness trade-offs to adapt to an antimicrobial and become resistant have not been explored so far in mycoplasmas. In other terms, what are the fitness costs of antimicrobial resistance on growth rate or on virulence? Are there a compensatory evolution and broader effects of genetic background? Does it impact on long-term evolutionary trends, as suggested by the epidemiological pattern of *M. bovis* in France? Genome-wide association studies, i.e., cumulative SNPs associated with patterns of low versus high MIC strains, could be a promising approach to explore.

## Figures and Tables

**Table 1:** List of clinically relevant species in human and animals, including the associated clinical signs and their prevalence worldwide.

Host	Mycoplasma Species	Clinical Signs or Syndrome	Geographical Prevalence
Humans	*M. genitalium*	Urethritis, cervicitis, pelvic inflammatory disease	Frequent, worldwide
	*M. pneumoniae*	Upper and lower respiratory tract infection	Frequent, worldwide
	*M. hominis*	Commensal of the urogenital tract (opportunistic pathogen)	Frequent, worldwide
	*U. parvum*, *U. urealyticum*	Commensal of the urogenital tract (opportunistic pathogen)	Frequent, worldwide
Cattle	*M. bovis*	Infectious enzootic bronchopneumonia, mastitis, arthritis, otitis	Frequent, worldwide
	*M. mycoides* subsp. *mycoides*	Contagious bovine pleuropneumonia	Scarce, Africa and Asia
Small ruminants	*M. putrefaciens*	Contagious agalactia	Regularly reported in Europe, particularly in Mediterranean regions, as well as the Middle East, Asia, North Africa and South America
	*M. agalactiae*		
	*M. mycoides* subsp. *capri*		
	*M. capricolum* subsp. *capricolum*		
	*M. capricolum* subsp. *capripneumoniae*	Contagious caprine pleuropneumonia	Scarce, Africa and Asia
	*M. ovipeumoniae*	Atypical pneumonia (facultative pathogen)	Infrequent, worldwide
Chickens, turkeys	*M. gallisepticum*	Chronic respiratory disease, infectious sinusitis	Frequent, worldwide
	*M. synoviae*	Subclinical respiratory tract infections, infectious synovitis, eggshell apex abnormality syndrome in laying-hen flocks (facultative pathogen)	Frequent, worldwide
Swine	*M. hyopneumoniae*	Enzootic pneumonia	Frequent, worldwide
	*M. hyorhinis*	Polyserositis, arthritis (facultative pathogen)	Frequent, worldwide
	*M. hyosynoviae*	Arthritis, polyarthritis (facultative pathogen)	Frequent, worldwide
	*M. suis*	Infectious Anaemia in Pigs, chronic immunosuppression	Frequent, worldwide

## Data Availability

Not applicable.
